# 5 CLABSI prevention with secure chat messaging

**DOI:** 10.1017/ash.2026.10462

**Published:** 2026-06-23

**Authors:** Emma Moore, Celina Santiago, Nicole Burton, Molly Kratz, Leah Seifu, Tristan McPherson, William Greendyke, Elise Mantell

**Affiliations:** 1 NYC Department of Health and Mental Hygiene; 2 NYC DOHMH; 3 New York City Department of Health and Mental Hygiene; 4 New York City Health Dept

## Abstract

**Background:** Feedback reports for medical providers can reduce antibiotic resistance by improving provider antimicrobial prescribing. In 2025, the New York City (NYC) Health Department created and sent antibiotic prescribing feedback reports to a subset of NYC-based primary care providers. **Method:** We procured 2019– application, a digital platform with antimicrobial stewardship resources. We emailed reports using Public Health Partners Connect, a communication management system. Only providers associated with a NYC address at the time of outreach, with a valid e-mail address, and who had not previously opted out of correspondence with the American Medical Association were included. **Result:** Of 716 sent reports, 51% (365) were to internal medicine physicians, 16% (114) to family medicine physicians, 14% (100) to physician assistants, 12% (84) to nurse practitioners, and 7% (53) to pediatricians. Of sent reports, 95% (677) were delivered, and 29% (194) of those recipients opened the report. Most who opened reports (83%) had high engagement (<10 seconds). Only one person opted out of receiving future emails, and no complaints were received in response. **Conclusion:** IQVIA claims data paired with OneKey provider email addresses enabled detailed and specific provider feedback reports on antibiotic prescribing. Including antibiotic appropriateness, duration, and spectrum of activity, rather than volume of antibiotics prescribed alone, allowed us to give providers more robust feedback than would be feasible using only publicly available data. The efficacy of these provider feedback reports could be evaluated with a subsequent data purchase and analysis.

